# Sprouting Angiogenesis in Human Pituitary Adenomas

**DOI:** 10.3389/fonc.2022.875219

**Published:** 2022-05-05

**Authors:** Jie Zhou, Yaomin Hu, Wende Zhu, Chuansheng Nie, Wenxiu Zhao, Alexander T. Faje, Kay E. Labelle, Brooke Swearingen, Hang Lee, E. Tessa Hedley-Whyte, Xun Zhang, Pamela S. Jones, Karen K. Miller, Anne Klibanski, Yunli Zhou, Roy J. Soberman

**Affiliations:** ^1^Neuroendocrine Unit, Massachusetts General Hospital and Harvard Medical School, Boston, MA, United States; ^2^Neurosurgery Department, Massachusetts General Hospital and Harvard Medical School, Boston, MA, United States; ^3^Biostatistics Center, Massachusetts General Hospital and Harvard Medical School, Boston, MA, United States; ^4^Department of Pathology (Neuropathology), Massachusetts General Hospital and Harvard Medical School, Boston, MA, United States; ^5^Nephrology Division, Massachusetts General Hospital and Harvard Medical School, Boston, MA, United States

**Keywords:** sprouting angiogenesis, angiogenic gene expression, angiogenesis inhibition, endothelial marker, Rb1 mice, VEGF inhibitor, cabozantinib, pituitary adenoma

## Abstract

**Introduction:**

Angiogenesis in pituitary tumors is not fully understood, and a better understanding could help inform new pharmacologic therapies, particularly for aggressive pituitary tumors.

**Materials and Methods:**

219 human pituitary tumors and 12 normal pituitary glands were studied. Angiogenic genes were quantified by an angiogenesis qPCR array and a TaqMan probe-based absolute qPCR. Angiogenesis inhibition in pituitary tumors was evaluated *in vitro* with the endothelial tube formation assay and *in vivo* in RbΔ19 mice.

**Results:**

71 angiogenic genes, 40 of which are known to be involved in sprouting angiogenesis, were differentially expressed in pituitary tumors. Expression of endothelial markers CD31, CD34, and ENG was significantly higher in pituitary tumors, by 5.6, 22.3, and 8.2-fold, respectively, compared to in normal pituitary tissue. There was no significant difference in levels of the lymphatic endothelial marker LYVE1 in pituitary tumors compared with normal pituitary gland tissue. Pituitary tumors also expressed significantly higher levels of angiogenesis growth factors, including VEGFA (4.2-fold), VEGFB (2.2), VEGFC (19.3), PGF (13.4), ANGPT2 (9.2), PDGFA (2.7), PDGFB (10.5) and TGFB1 (3.8) compared to normal pituitary tissue. Expression of VEGFC and PGF was highly correlated with the expression of endothelial markers in tumor samples, including CD31, CD34, and ENG (endoglin, a co-receptor for TGFβ). Furthermore, VEGFR inhibitors inhibited angiogenesis induced by human pituitary tumors and prolonged survival of RbΔ19 mice.

**Conclusion:**

Human pituitary tumors are characterized by more active angiogenesis than normal pituitary gland tissue in a manner consistent with sprouting angiogenesis. Angiogenesis in pituitary tumors is regulated mainly by PGF and VEGFC, not VEGFA and VEGFB. Angiogenesis inhibitors, such as the VEGFR2 inhibitor cabozantinib, may merit further investigation as therapies for aggressive human pituitary tumors.

## Introduction

Approximately 15% of intracranial neoplasms are pituitary tumors, the vast majority of which are benign and slow-growing. Microadenomas (tumors < 1 cm) that are not hormone-secreting (i.e., nonfunctioning) can be monitored without intervention, whereas hormone-secreting tumors, except for prolactinomas for which dopamine agonist therapy is usually first-line therapy) are generally treated with surgery. Surgery is also the primary treatment modality for macroadenomas (tumors >1 cm), except for prolactinomas (1). Approximately 10% of pituitary tumors recur following surgery and are treated with repeat surgery, pharmacologic therapy and/or radiation (2). A very small percentage become locally invasive and/or metastasize despite standard therapies, including radiation (3, 4), and there are few effective therapies for these aggressive pituitary tumors. Temozolomide is first-line therapy for very aggressive tumors, including pituitary carcinomas, but escape usually occurs. Case reports suggest that checkpoint inhibitor therapy may have activity against a subset of such tumors, but preliminary reports indicate that responses are mixed. Therefore, new approaches are needed for the treatment of aggressive adenomas unresponsive to conventional therapies.

Multiple angiogenesis inhibitors targeting angiogenesis signaling pathways have been approved by the FDA to treat a wide range of human cancers (5, 6). Many more are currently under development or in clinical trials (5, 7). However, none of these drugs are approved for the treatment of pituitary tumors. To date, fewer than 20 patients with aggressive pituitary tumors have been reported to have been treated with antiangiogenic therapy, many following treatment with temozolomide (8, 9). Bevacizumab (Avastin^®^), a monoclonal antibody against VEGFA, has been most frequently reported in this context (N=11) (8, 9). Use of Sunitinib (Sutent^®^), a pan kinase inhibitor targeting VEGF receptors and PDGF receptors, and Apatinib, mainly targeting VEGFR2, have been reported in one case each (8). The treatment outcomes in these 13 patients ranged from a complete response to progressive disease (8). We suspect that one likely reason for the infrequent usage of angiogenesis inhibitors in pituitary tumor patients is the lack of data on angiogenesis in these tumors.

Angiogenesis is the process of forming new blood vessels from pre-existing ones (10) and has been implicated as a mechanism responsible for driving tumor growth, which is limited without neovascularization (11). Tumor vascularization can occur through vasculogenesis, vasculogenic mimicry, and intussusception, with endothelial sprouting identified as particularly important (12, 13). Vascular endothelial growth factors (VEGFs) are the master regulators of this process (14), which involves multiple signaling pathways (15) (16). The few studies examining angiogenesis specifically in pituitary tumors have demonstrated contradictory results. In contrast to the enhanced angiogenesis observed in many malignant tumors, Turner et al. demonstrated a lower density of blood vessels in benign pituitary tumors than in normal pituitary tissue, though macroadenomas demonstrated greater vascularity than microadenomas (17). In addition, most of the pituitary tumor angiogenesis literature focuses on microvessel density and endothelial markers, with McCabe et al. in addition observing an approximately 3-fold increase in VEGFA expression in nonfunctioning adenomas (NFAs) compared to normal pituitary tissue (18). We wanted to build on this literature by examining angiogenesis signaling pathways in pituitary tumors.

In this study, we used the TaqMan^®^ probe-based absolute quantitative PCR method to assess expression of multiple growth factors from the major angiogenic signaling pathways in a large cohort of human pituitary tumors. We also examined expression of several vascular endothelial marker genes. Furthermore, we determined that VEGF signaling plays a major role in pituitary tumor angiogenesis and that blocking VEGF signaling significantly extended survival in a pituitary tumor mouse model.

## Materials and Methods

### Human Subjects and Tumor Specimens for Gene Expression Assays

We studied 219 pituitary tumors: 151 clinically nonfunctioning adenomas (NFAs), 39 GH-secreting, 13 PRL-secreting, and 16 ACTH-secreting tumors (**Table 1**). Twenty-seven of these tumor samples were used in our previous experiments examining immune checkpoint molecules, which is unrelated to this study (19). Tumors from 132 males and 87 females were included. Patients ranged from 19 to 83 years, with a mean (SD) of 52 (16) years. Pituitary tumor samples were collected immediately following surgery and promptly used for primary tissue culture or stored in liquid nitrogen. Twelve control normal anterior pituitary glands were obtained from patients who died of non-endocrine causes. All glands were sampled for microscopy before donating the remainder of the unfixed tissue for research. They were considered to be normal if no abnormalities were observed. These tissues were fixed in RNAlater (Thermo Fisher Scientific, Waltham, MA) and stored at -80°C. The study was approved by the Mass General Brigham Institutional Review Board.

**Table 1:** Clinical Characteristics.

Tumor Types	Number of Patients			Age at Surgery (mean ± SD)	Tumor Size^b^(cm)(mean ± SD)	Ki67 index(%)(mean ± SD)
	Total	Male	Female			
All tumors	219	132	87	53 ± 16	2.3 ± 1.0	1.9 ± 1.6
Clinically non-functioning	151	105	46	57 ± 14	2.5 ± 0.9	1.7 ± 1.8
ACTH-producing	16	1	15	48 ± 13^c^	1.3 ± 0.8^c^	3.1 ± 2.7^c^
GH-producing	39	22	17	45 ± 15^c^	2.0 ± 1.0^c,d^	2.1 ± 1.6
PRL-producing^a^	13	6	7	34 ± 11^c,d,e^	2.1 ± 0.8^d^	2.1 ± 2.1

a PRL-producing tumors were primarily resected from patients who were intolerant to medical treatment or had tumors that were resistant to medical treatment.

b Tumor size at baseline, measured in the largest dimension. Comparisons were performed using a one-way ANOVA multiple comparison test. ^c^p < 0.05 vs NFA; ^d^p < 0.05 vs ACTH-producing adenoma; ^e^p < 0.05 vs GH-producing adenoma.

### RNA Extraction and Reverse Transcription

Total RNA was extracted using Qiagen RNeasy^®^ Mini Kits (#74104) following the manufacturer’s instructions (Qiagen, Valencia, CA). Eluted RNA was treated with Qiagen RNase-free DNase and purified with Qiagen RNeasy MinElute Cleanup kit (#74204) following the manufacturer’s instructions. Reverse transcription was performed with a specific amount of total RNA (ranging from 0.4 to 0.9 μg) using the ProtoScript^®^ First Strand cDNA Synthesis Kit per the manufacturer’s instructions (New England Biolabs, Ipswich, MA). The final volume of each RT reaction was 50 μl.

### Angiogenesis RT^2^ Profiler PCR Array Analysis

Human Angiogenesis RT^2^ Profiler PCR array kits were used (Qiagen). RNA extracted from human pituitary tumors was pooled by combining 0.5 μg per sample. The pooled RNA contained equal quantities of RNA from 207 tumor samples, including 140 NFAs, 38 GH, 16 ACTH, and 13 PRL-secreting tumors. Reverse transcriptions were carried out with 1 μg pooled RNA per reaction as described above. The PCR with the array was performed following the protocol provided by Qiagen. PCR data were analyzed using Qiagen web-based PCR Array Data Analysis Software. The results from three experiments are presented as fold changes in tumor tissue compared to normal control tissue. P values < 0.05 were considered statistically significant.

### Absolute TaqMan^®^ Probe-Based Quantitative RT-PCR

Real-time PCR was performed using TaqMan^®^ gene expression assays (Thermo Fisher Scientific, Waltham, MA). Briefly, real-time PCR was performed in triplicate in 20 μl per well containing 1 μl of RT reaction, 1 μl of specific TaqMan^®^ probes, and 10 μl of TaqMan^®^ Universal PCR Master Mix (Thermo Fisher Scientific, Waltham, MA). PCR was run on an Applied Biosystems^®^ 7500 FAST Real Rime PCR System at 50°C for 2 min, followed by 95°C for 10 min, followed by 40 cycles at 95°C for 15 sec, and 60°C for 1 min. PCR data were analyzed by setting baseline auto and thresholds to 0.065.

To quantify gene expression, standard curves for TaqMan^®^ probes were generated using linearized plasmid DNAs as templates. Real-time PCR was performed in quadruplicate containing 10^8^, 10^7^, 10^6^, 10^5^, 10^4^, 10^3^, 10^2^, and 10 molecule copies of template DNA. Standard curves were generated in Excel using the XY-plot function, where x represents the log of copy number and y represents the cycle threshold (Ct) value. The y-intercept, slope, and coefficient of determination were computed. The amplification efficiency (€) was calculated using the following formula: [10^(-1/slope)^ – 1] x 100. Because one-fiftieth of the volume of an RT reaction was used for each PCR reaction, the expression of specific genes in tissue samples was calculated as 10^[–A - Intercept)/Slope]^ x 50/RNA_Input_, where A is the average of Ct values generated from triplicate PCR reactions and RNA_Input_ the amount of total RNAs in μg used in the RT reaction. The resultant values are the numbers of transcript copies per μg total RNA. The genes, TaqMan^®^ probes, plasmid DNA templates, and their corresponding qPCR standard curve parameters are provided in **Table 2**.

**Table 2:** Genes, TaqMan^®^ Probes, and qPCR Standard Curve Performance.

Gene Symbol	Accession #	Gene Full Name	TaqMan^®^ Assay ID	Standard Curve^a^			
				Template	I	€	R^2^
ANGPT1	NM_001146.4	angiopoietin 1	Hs00919202_m1	pGEM-ANGPT1^c^	36.5	93.5%	0.998
ANGPT2	NM_001147.2	angiopoietin 2	Hs00169867_m1	pCR4-TOPO-ANGPT2^b^	36.4	97.3%	1.000
CD31	NM_000442.4	platelet and endothelial cell adhesion molecule 1	Hs01065279_m1	pCMV-Sport6-PECAM1^b^	35.2	95.7%	1.000
CD34	NM_001025109.1	CD34 molecule	Hs02576480_m1	pCMV-Sport6.1-CD34^b^	36.7	92.5%	0.999
ENG	NM_001114753.2	endoglin	Hs00923996_m1	pOTB7-ENG^b^	36.8	96.8%	1.000
FN1	NM_212482.2	fibronectin 1	Hs01549976_m1	pCR-XL-TOPO-FN1^b^	36.8	94.7%	0.999
HIF1A	NM_001530.3	hypoxia inducible factor 1 alpha subunit	Hs00153153_m1	pGEM-HIF1A^c^	36.6	94.6%	1.000
KDR	NM_002253.2	kinase insert domain receptor	Hs00911700_m1	pMD18-VER2^c^	36.4	92.8%	0.999
LYVE1	NM_006691.3	lymphatic vessel endothelial hyaluronan receptor 1	Hs01119300_g1	pDNR-LIB-LYVE1^b^	35.3	96.4%	0.999
PDGFA	NM_002607.5	platelet derived growth factor subunit A	Hs00234994_m1	pCMV6-Entry-PDGFA^d^	36.4	95.9%	1.000
PDGFB	NM_002608.3	platelet derived growth factor subunit B	Hs00966522_m1	pCMV-Sport6-PDGFB^b^	36.3	98.6%	1.000
PGF	NM_001207012.1	placental growth factor	Hs00182176_m1	pOTB7-PGF^b^	36.6	98.8%	1.000
TEK	NM_000459.4	TEK receptor tyrosine kinase	Hs00945150_m1	pCMV-Sport6-TEK^d^	36.0	94.7%	0.998
TGFB1	NM_000660.6	transforming growth factor beta 1	Hs00998133_m1	pCMV-Sport6-TGFB1^b^	35.4	92.6%	0.998
TGFB2	NM_001135599.3	transforming growth factor beta 2	Hs00234244_m1	pMD18-TGFB2^d^	36.0	98.9%	1.000
VEGFA	NM_001025366.2	vascular endothelial growth factor A	Hs00900055_m1	pCMV-Sport6-VEGFA^a^	36.6	94.6%	0.999
VEGFB	NM_001243733.1	vascular endothelial growth factor B	Hs00173634_m1	pCMV6-entry-VEGFB^d^	35.5	93.7	1.000
VEGFC	NM_005429.4	vascular endothelial growth factor C	Hs01099203_m1	pCMV-Sport6-VEGFC^b^	35.8	96.5%	1.000
GAPDH	NM_002046.5	glyceraldehyde-3-phosphate dehydrogenase	Hs03929097_g1	pCMV-Sport6-GAPDH^b^	35.8	96.5%	1.000

^a^ I is the y-intercept of the XY plots, where y is the cycle threshold (Ct) and x the log template amount; € is the amplification efficiency calculated as €=[10^(-1/slope)^]-1; R^2^ is the coefficient of determination. Template DNAs, ^b^obtained from GE Dharmacon; Lafayette, CO, ^c^from Sino Biological; Beijing, China; ^d^from OriGene Technologies; Rockville, MD.

### Detection of Protein by ELISA

To detect growth factors released by pituitary tumors, fresh tumor tissue was weighed and cultured with 2 ml DMEM/F12 medium containing 10% FBS. Forty-eight hours after incubation, medium supernatants were collected and stored as 100 μl aliquots at -80°C. ANGPT2 (Cat. #DANG20), PGF (#DPG00), and VEGFC (#DVEC00) were assayed using Quantikine^®^ ELISA kits (R&D systems, Minneapolis, MN) per the manufacturer’s instructions. The total quantity of each growth factor detected in 2 ml of medium was divided by the tissue weight to generate pg of each growth factor per mg tissue. To detect endothelial marker proteins, a small piece of tumor tissue was weighed, minced, and homogenized in 350 μl of RIPA lysis buffer containing a protease inhibitor cocktail from Sigma Aldrich (P8340, St. Louis, MO). The lysates were diluted with PBS and assayed with G-Biosciences ELISA kits for CD31 (#501482738) and CD34 (#501484437) (Thermo Fisher Scientific, Waltham, MA). ENG was assayed with Quantikine^®^ ELISA kits (#DNDG00) (R&D systems, Minneapolis, MN). The total quantity of each endothelial marker detected in 350 μl of lysates was divided by the weight of the tissue to generate pg per mg tissue.

### Endothelial Cell Tube Formation Assay

Human umbilical vein endothelial cells (HUVEC) were obtained from Lonza (Walkersville, MD) and maintained in the Lonza EBM™ basal medium plus SingleQuot™ growth factors (CC-4133) following the manufacturer’s instructions. To obtain medium conditioned by human pituitary tumors, tumor tissue was washed with PBS, minced, and incubated with serum-free DMEM/F12 medium at 37°C and 5% CO_2_ for 24 hours. The medium was collected and filtered through 0.45 μm filters before use. To ensure that the assay was not affected by the DMEM/F12 media, we cultured HUVEC cells with this media supplemented with the aforementioned growth factors for up to three days and observed no adverse effect on cell proliferation.

The tube formation assay was performed as described previously (20). Briefly, Matrigel^®^ matrix (Corning Life Science, Tewksbury, MA) was added to 24-well plates (300 ul per well) and incubated in a 5% CO2, 37°C incubator for one hour. HUVECs (1.5 × 10^5^) suspended in 300 μl of medium conditioned by human pituitary tumor tissue were seeded to the Matrigel^®^ coated plates. To determine the effect of inhibition of the VEGF pathway on tube formation, conditioned media were supplemented with DMSO, regorafenib (S1178, Selleck Chemicals, Houston, TX), or cabozantinib (S1119) at a final concentration of 5 μM. After a 16-hour additional incubation, tube formation was photographed under an inverted light microscope. Five images per well were captured. Tube lengths and branching points were quantified on the images using the online WimTube program (21).

### Pituitary Tumor Mouse Model and Cabozantinib Treatment

The mice were housed at the animal facility of the Center for Comparative Medicine at Massachusetts General Hospital. The study was approved by the MGH Institutional Animal Care and Use Committee (IACUC).

*Rb1^tm2Brn^* mice carrying a floxed exon 19 of the *Rb1* gene (also known as Rb1*fl/fl*) were obtained from the Jackson Laboratory (Bar Harbor, ME) (22). RbΔ19 mice were created by crossing *Rb1^tm2Brn^* mice with CMV-Cre (BALB/c-Tg(CMV-cre)1Cgn/J, the Jackson Laboratory) and maintained as heterozygotes on C57BL/6 background. Mice used in the experiments were at least 8^th^ generations subsequent to backcrossing with C57BL/6 mice. Thirty-two heterozygous RbΔ19 mice, as well as 27 wild-type (WT) littermates, were monitored. Mice were euthanized when they became moribund. The cause of death was determined by necropsy examination. Twenty-five RbΔ19 mice euthanized had large pituitary tumors. Other than thyroid tumors in eight mice, no apparent tumors or other significant anomalies were observed. For these mice, large pituitary tumors were the cause of death. Four RbΔ19 mice grew large neck masses affecting their breathing and had to be euthanized according to our IACUC policy. Necropsy examination showed that they had no or very small pituitary tumors. Three RbΔ19 mice died unexpectedly. No tumors were found in their pituitary glands. The cause of death was unknown. These mice were not included in the final survival analysis. Of the 27 WT littermates, only one died. This mouse had no pituitary tumor, and the cause of death was unknown. The survival curves were plotted, and median survival was determined using Prism 9 software.

Cabozantinib (XL184) (Cab), a potent inhibitor of VEGF receptor 2 (23), was obtained from Selleck Chemicals (Houston, TX). A suspension in sterile PBS of a final concentration of 5 mg/ml was prepared. The drug was administered once daily to mice *via* oral gavage at a concentration of 15 or 30 mg/kg body weight (designated as Cab-15 and Cab-30, respectively) for 14 days. After a 14-day break, the drug regimen was resumed for another 14 days. PBS was administered to another group of mice as controls. The number of mice receiving treatment in each group was as follows: 28 mice for the control PBS group, 26 for the cab-15 group, 28 for the cab-30 group, and 10 for the WT mice group, which was administered 30 mg Cab/kg body weight. The health of these mice was monitored, and they were euthanized when they reached a moribund state. After euthanasia, the mice underwent necropsy to determine the cause of death. Mice who died of causes unrelated to pituitary tumors were not included in the final analysis.

In the PBS group, five mice were removed from experiments because of large neck masses, which caused extreme difficulty with breathing. These neck masses were thyroid tumors. Necropsy revealed that these mice had very tiny pituitary tumors or no apparent pituitary tumors. In the Cab-15 group, one mouse was removed from the experiment because of severe conjunctivitis, one was removed because of a large neck mass, and two were removed due to sudden death soon after the treatment started, likely due to injuries caused by oral gavage. Necropsy revealed these mice had tiny pituitary tumors only. In the Cab-30 group, four mice were removed from experiments because of large neck masses. The rest of the mice in these groups ultimately became moribund. The moribund state was caused by large pituitary tumors in all of these cases, and no apparent tumors were identified in other tissues.

### Statistical Analyses

Data were analyzed using GraphPad Prism 9 software. The one-way ANOVA multiple comparison test was used to compare clinical characteristics. The Kruskal-Wallis nonparametric one-way ANOVA test was used to compare data from endothelial cell tube formation assays and gene expression levels between tumor groups and controls. Pearson’s correlations were applied to determine relationships between expression levels among individual genes. The Kaplan-Meir method was applied to construct survival curves, and the log-rank (Mantel-Cox) test was used for between groups comparison. P values < 0.05 were considered significant.

## Results

### Expression of Angiogenic Genes in Human Pituitary Tumors Determined by the RT² Profiler PCR Array

**Figure 1:**
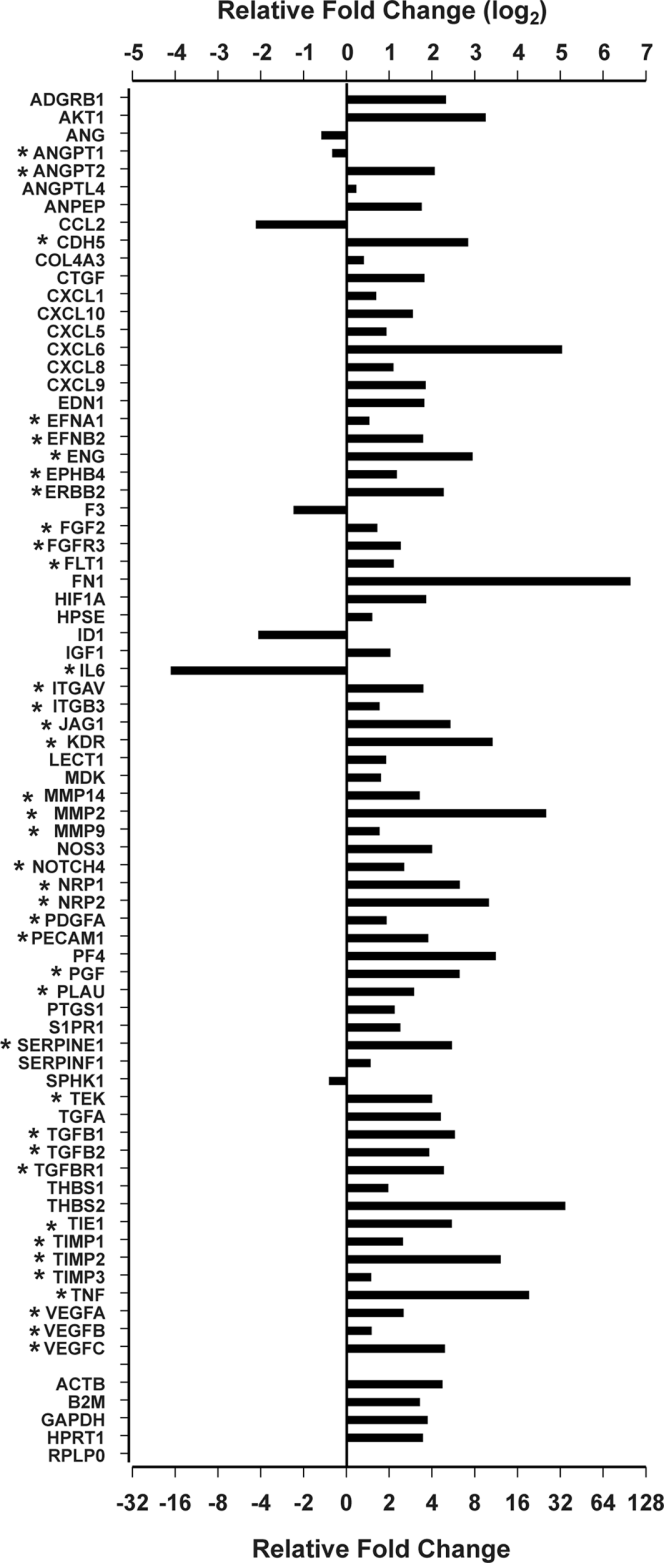
Differentially expressed angiogenesis genes by the Qiagen RT^2^ Profiler PCR Array. *Genes involved in sprouting angiogenesis.

To evaluate the expression of angiogenesis-related genes in human pituitary tumors, we used the Qiagen human angiogenesis RT² profiler PCR array to evaluate RNA pooled from 207 pituitary tumor samples. RNA pooled from 12 normal human pituitary glands was used as the control. After PCR, we chose the RPLP0 gene as the internal control for Ct normalization following the manufacturer’s instructions because the difference between Ct values from tumor plates and control plates was less than 1. The PCR array listed 84 angiogenesis-related genes (**Supplementary Table 1**). Four genes (IFNA1, IFNG, LEP, and PLG) were excluded from analysis because their PCR Ct values were greater than 32 for both tumor and control samples, indicating that the expression of these genes was extremely low and not reliable for comparison analysis. Among the remaining genes, seventy-one were expressed differentially between pituitary tumor and normal pituitary gland tissue with p values less than 0.05 (**Figure 1** and **Supplementary Table 1**). Sixty-four genes were upregulated, whereas seven were downregulated.

More than half of these differentially expressed genes (n=39) are known to regulate sprouting angiogenesis (14, 15, 24–28) (**Figure 1**). Most of these are components of signal transduction pathways regulating sprouting angiogenesis: VEGF pathway (VEGFA, VEGFB, VEGFC, PGF, KDR, FLT1, EFNB2, ITGAV, ITGB3, CDH5, EFNB2, NRP1, and NRP2) (26, 29), the angiopoietin-Tie pathway (ANGPT1, ANGPT2, and TEK) (30), the Notch pathway (NOTCH4 and JAG1) (27), the PDGF pathway (PDGFA) (31), and the TGFβ pathway (TGFB1, TGFB2, TGFBR1, and ENG) (32). Recently, Zegeye et al. reported that IL-6 inhibits sprouting angiogenesis by trans-signaling (33). We found that IL-6 expression was downregulated 17.1-fold in human pituitary tumor tissue compared to normal pituitary tissue (**Figure 1**). In addition, the expression of two well-known endothelial marker genes, *ENG* and *PECAM1* (34, 35) was increased in tumor tissue by 3.72 and 6.67-fold, respectively (**Figure 1**). The gene with the most elevated expression was *FN1*, which was nearly 100-fold higher in human pituitary tumor tissue than in normal pituitary samples (**Figure 1**). Products of the *FN1* gene are essential components of newly formed blood vessels (36). These data indicate that human pituitary tumors contain a more active angiogenesis program than normal pituitary tissue.

### Expression of Vascular Endothelial Marker Genes in Human Pituitary Tumors

The PCR array showed that the expression of four out of five internal reference genes was approximately 4-fold higher in human pituitary tumor tissue than in normal pituitary tissue (**Figure 1** and **Supplementary Table 1**), suggesting that a relative quantitative PCR method would underestimate gene expression after normalization against these genes. Therefore, we employed an absolute quantitative PCR method to quantify the expression of these genes, using external DNA as a reference standard.

**Figure 2:**
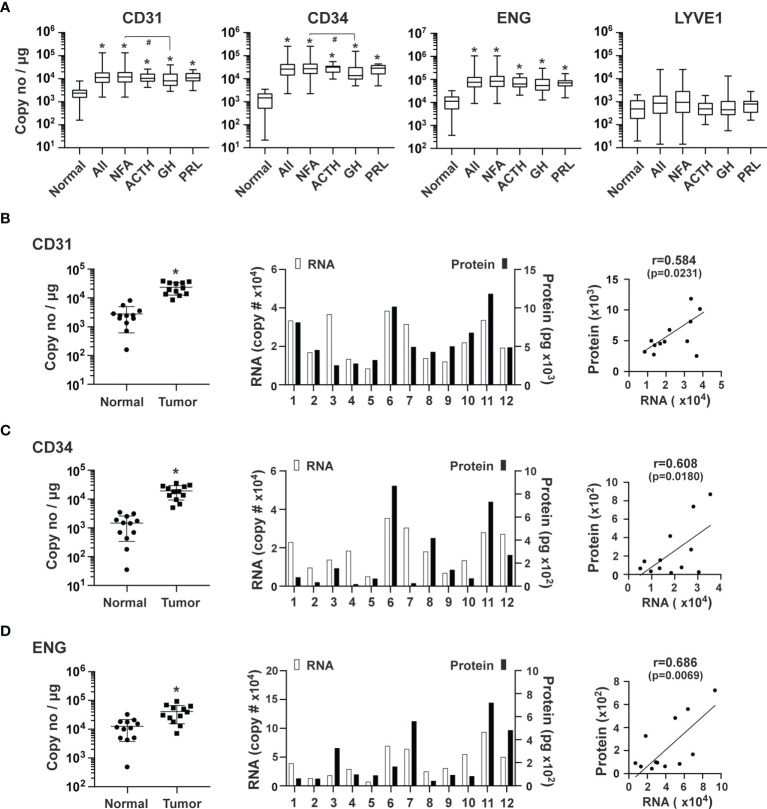
Expression of endothelial markers in human pituitary tumors. **(A)** Expression of the indicated genes was quantified by the TaqMan^®^ probe-based qPCR. A total of 207 human pituitary tumors, including 140 NFAs, 38 GH-producing, 16 ACTH-producing and 13 PRL-producing tumors, and 12 normal pituitaries were assessed. *p < 0.05 vs normal pituitaries; #p < 0.05 vs NFA. **(B)** CD31, **(C)** CD34 and **(D)** ENG: Correlation of their RNA and protein expression in an additional 12 pituitary tumors, including 11 NFA and 1 GH-secreting tumors. *Left panel*, RNA levels of endothelial markers in pituitary tumors and normal pituitary tissue. *p < 0.05 vs normal pituitaries. *Middle panel*, RNA and protein levels of individual pituitary tumors. RNA levels (open bars) are indicated on the left y-axis as copies of transcript/μg total RNA. Protein levels (solid bars) are indicated on the right y-axis as pg/mg of tumor tissue. *Right panel*, correlation between RNA levels determined by qPCR and protein levels determined by ELISA.

We quantified the expression of *ENG* and *PECAM* and also determined the expression levels of CD34 mRNA, which encodes a glycoprotein expressed in early, mature, and progenitor endothelial cells (37). We also quantified the expression of mRNA coding of LYVE1, a marker of lymphatic endothelial cells (38). The RNA transcripts detected in pituitary tumors for CD31, CD34 and ENG were 1.6 x 10^4^, 3.3 x 10^4,^ and 1.0 x 10^5^ copies/μg total RNA, respectively. Compared with normal pituitary tissue, RNA levels of CD31, CD34, and ENG were increased in pituitary tumors by 5.6 (p<0.0001), 22.6 (p<0.0001), and 8.2 (p<0.0001) fold, respectively (**Figure 2A**). In contrast, there were 1.9 x 10^3^ copies/μg total RNA of LYVE1, which was not significantly different from that of controls (normal pituitary) (p>0.9999) (**Figure 2A**). These data indicate that the number of vascular endothelial cells, but not lymphatic endothelial cells, is significantly greater in all types of pituitary tumors than in normal pituitary tissue.

Because mRNA levels may not truly reflect their respective protein levels, we measured protein levels of CD31, CD34, and ENG in 12 additional pituitary tumors using ELISA assays. mRNA levels as determined by qPCR in these tumors were significantly higher than in normal pituitary tissue (**Figures 2B–D**, *left panels*). Their respective proteins were readily detected (**Figures 2B–D**, *middle panels*) and correlated well with mRNA levels (**Figures 2B–D**, *right panels*), suggesting that the mRNA levels we measured accurately reflect protein levels.

### Expression of Angiogenic Growth Factors in Human Pituitary Tumors

**Figure 3:**
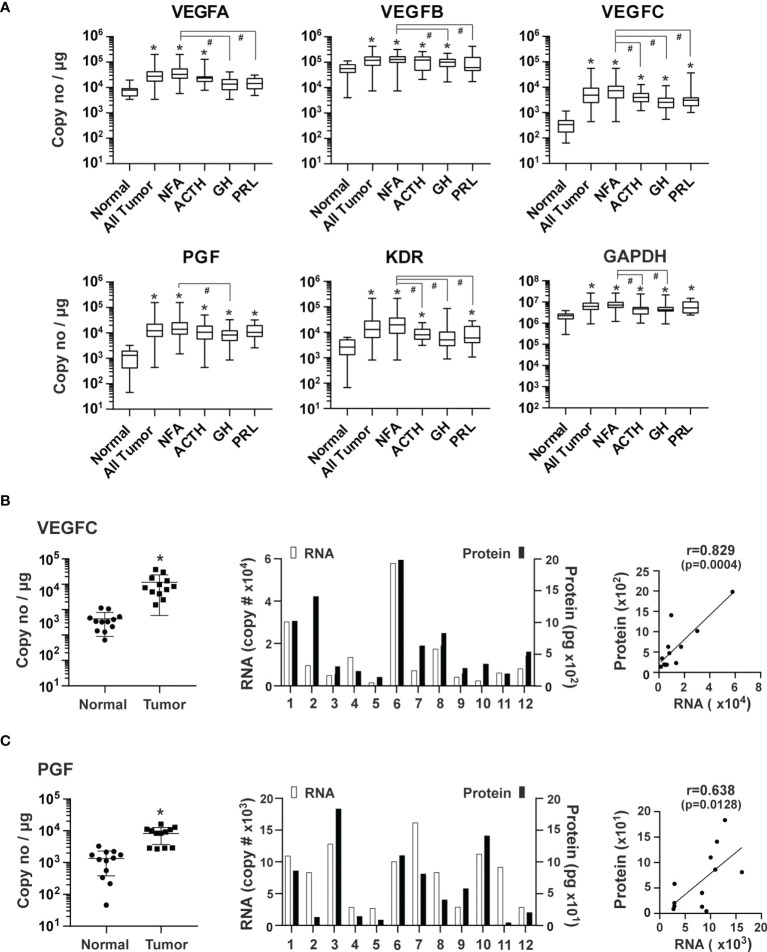
Expression of VEGF family of growth factors in human pituitary tumors. **(A)** Expression of indicated genes was quantified by the TaqMan^®^ probe-based qPCR. Normal pituitary tissue, n = 12; NFA, n = 140; ACTH, n = 16; GH, n = 38; PRL, n = 13. *p < 0.05 vs normal pituitary tissue; #p < 0.05 vs NFA. **(B)** VEGFC and **(C)** PGF, Correlation of RNA and corresponding protein expression in an additional 12 pituitary tumors, including 11 NFA and 1 GH-secreting tumors. *Left panel*, RNA levels of endothelial markers in pituitary tumors and normal pituitary tissue. *p < 0.05 vs normal pituitary tissue. *Middle panel*, RNA and protein levels of individual pituitary tumors. RNA levels (open bars) were indicated on the left y-axis as copies of transcript/μg total RNA. Protein levels (solid bars) were indicated on the right y-axis as pg/mg of tumor tissue. *Right panel*, correlation between RNA levels determined by qPCR and protein levels determined by ELISA.

The VEGF family of growth factors are master regulators of sprouting angiogenesis, as they stimulate proliferation and guide the migration of endothelial cells (24, 25, 27). VEGFA, VEGFB, VEGFC, and PGF, as well as their receptor, VEGFR2, also known as the kinase insert domain receptor (KDR), were examined. The genes with the highest to lowest levels of expression were: VEGFB (1.1 x 10^5^ copies/μg total RNA), VEGFA (3.5 x 10^4^), PGF (1.8 x 10^4^), and VEGFC (8.3 x 10^3^), respectively. Compared with normal pituitary tissue, genes with the highest to lowest expression levels in pituitary tumor tissue were VEGFC (19.3-fold, p<0.0001), PGF (13.4-fold, p<0.0001), VEGFA (4.2-fold, p<0.0001) and VEGFB (2.2-fold, p=0.002) (**Figure 3A**). Moreover, elevated expression in all types of pituitary tumors was only observed with VEGFC and PGF. In contrast, VEGFA expression was increased in NFA and ACTH-secreting tumors but not in GH and PRL-secreting tumors (**Figure 3A**). KDR expression was 7.8-fold (p<0.0001) higher in all types of pituitary tumors combined than in normal pituitary tissue (**Figure 3A**). As a non-angiogenesis gene control, GAPDH expression was determined to be 7.6 x 10^6^ copies/μg total RNA. Compared to the normal pituitary tissue, GAPDH expression was higher by 3.5-fold (p<0.0001) in tumors (**Figure 3A**), which confirmed the PCR array findings.

We next measured primary pituitary tumor production of PGF and VEGFC proteins. We cultured fresh tissue from 12 tumors for 48 hours and assessed PGF and VEGFC in culture media using ELISA. PGF and VEGFC proteins were readily detected in the culture medium (**Figure 3B**). Expression levels of mRNA and protein correlated well (**Figure 3B**).

**Figure 4:**
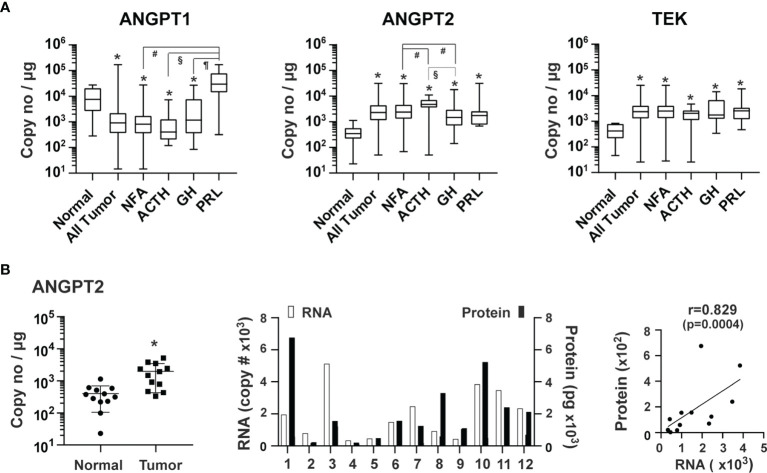
Expression of angiopoietins and their receptor, TEK, in human pituitary tumors. **(A)** RNA expression of ANGPT1, ANGPT2 and TEK was quantified by TaqMan^®^ probe-based qPCR. Normal pituitary tissue, n = 12; NFA, n = 140; ACTH, n = 16; GH, n = 38; PRL, n = 13. *p < 0.05 vs normal pituitary tissue; #p < 0.05 vs NFA; §p < 0.05 vs ACTH-secreting tumors; ¶p < 0.05 vs GH-secreting tumors. **(B)** Correlation of ANGPT2 RNA in tumor tissue and angiopoietin 2 released by the corresponding tumors. A total of 12 pituitary tumors, including 11 NFA and 1 GH-secreting tumors were assessed. *Left panel*, RNA levels of endothelial markers in pituitary tumors and normal pituitary tissue. *p < 0.05 vs normal pituitary tissue. *Middle panel*, RNA and protein levels of individual pituitary tumors. RNA levels (open bars) are indicated on the left y-axis as copies of transcript/μg total RNA. Protein levels (solid bars) are indicated on the right y-axis as pg/mg of tumor tissue. *Right panel*, correlation between RNA levels determined by qPCR and protein levels determined by ELISA.

Angiopoietin-Tie signaling regulates the homeostasis of blood vessels (5, 30). Angiopoietin 1 (ANGPT1) and 2 (ANGPT2) regulate angiogenesis *via* their common TEK tyrosine kinase receptor (TEK, also known as TIE2) (30, 39). ANGPT1, which is mainly produced from perivascular cells, such as pericytes and vascular smooth muscle cells, activates TEK resulting in endothelial cell survival and stabilization (30). ANGPT1 over-expression inhibits angiogenesis (40). ANGPT2, highly expressed in endothelial tip cells (41, 42), promotes angiogenesis in conjunction with VEGF by antagonizing ANGPT1 (30). The overall expression of ANGPT1 was 5.4 x 10^3^ copies/μg total RNA in pituitary tumor tissue. Compared with normal pituitary tissue, the overall expression of ANGPT1 was significantly lower in pituitary tumor tissue by 2.1-fold (p=0.0043) (**Figure 4A**). The ANGPT1 downregulation was observed in all types of tumors except for PRL-producing tumors. In contrast, expression of ANGPT2 (3.6 x 10^3^ copies/μg total RNA) and TEK (3.4 x 10^3^) was significantly elevated in all types of pituitary tumors with an overall increase of 9.2 (p<0.0001) and 7.3-fold (p<0.0001), respectively (**Figure 4A**). The ANGPT2 protein was also detected in the culture medium of primary pituitary tumor tissue, and its protein levels correlated strongly with their corresponding RNA levels (**Figure 4B**).

**Figure 5:**
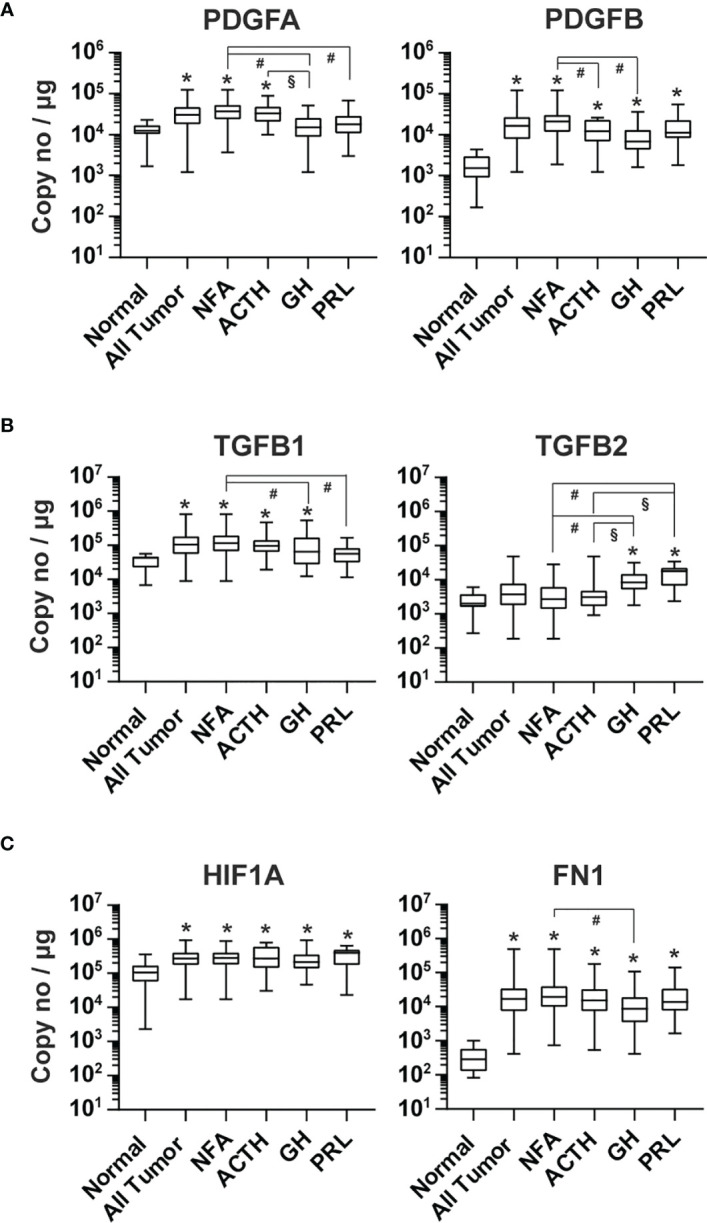
Expression of other angiogenic growth factors in human pituitary tumors. RNA of **(A)** PDGFA and PDGFB, **(B)** TGFB1 and TGFB2, and **(C)** HIF1A and FN1 were quantified by TaqMan^®^ probe-based qPCR. Normal pituitary tissue, n = 12; NFA, n = 140; ACTH, n = 16; GH, n = 38; PRL, n = 13. *p < 0.05 vs normal pituitaries; #p < 0.05 vs NFA; §p < 0.05 vs ACTH-producing tumors.

The PDGF and TGFβ signaling pathways regulate blood vessel maturation (43, 44). The expression of PDGFA, PDGFB, TGFB1 and TGFB2 in pituitary tumor tissue was respectively 3.5 x10^4^, 2.0 x 10^4^, 1.3 x 10^5^ and 6.6 x 10^3^ copies/μg total RNA (**Figure 5A**). PDGFB expression in all types of pituitary tumors was, on average, 10.5-fold (p<0.0001) greater than that of normal pituitary tissue (**Figure 5A**). Significantly higher PDFA expression was only detected in NFA and ACTH-secreting tumors compared with normal pituitary tissue. TGFB1 was significantly higher in NFA (4.2-fold, p<0.0001), ACTH-secreting tumors (3.5-fold, p=0.0017), and GH-secreting tumors (2.96-fold, p=0.007), but not in PRL-secreting tumors (**Figure 5B**) compared with normal pituitary tissue. Conversely, TGFB2 expression was significantly higher in GH-secreting (4.2-fold, p<0.0001) and PRL-secreting tumor tissue (6.1-fold, p<0.0001), but not in NFA and ACTH-secreting tumor tissue (**Figure 5B**), than in normal pituitary tissue.

The alpha subunit of hypoxia-induced transcription factor 1 (HIF1A) is known to activate the expression of PGF, PDGFB, VEGFA, and VEGFC (45, 46). RNA levels of HIF1A were significantly higher in all tumor types compared with normal pituitary tissue, with an overall elevation of 2.4-fold (p=0.0004) (**Figure 5C**).

We also determined the expression of *fibronectin 1* (*FN1*) in pituitary tumor tissue. PCR products of *FN1* containing EDA and EDB domains are mainly found in newly formed blood vessels and overexpressed in many human tumors (47). We found that the FN1 transcript level in human pituitary tumors was 3.4 x 10^4^ copies/μg total RNA, which was 89.6-fold (p<0.0001) that observed in normal pituitary tissue and significantly elevated in every tumor type (**Figure 5C**).

Sixteen of the 19 genes examined by absolute quantitative PCR in individual samples were included in the RT² Profiler PCR Array. The fold changes in pituitary tumors compared to normal pituitary gland tissue by these two methods correlated strongly (R=0.9815, p<0.0001). These data validate the RT² Profiler PCR Array findings and, more importantly, support the hypothesis that angiogenesis is enhanced in human pituitary tumors.

### Correlation of Vascular Endothelial Markers and Angiogenic Growth Factors in Human Pituitary Tumors

We calculated the expression ratio between endothelial markers and the lymphatic endothelial marker LYVE1 based on data in **Figure 2** to determine the contribution of vascular endothelial cells and lymphatic endothelial cells to the increase in endothelial marker expression in pituitary tumor tissue. In normal pituitary tissue, the expression ratios of CD31, CD34, and ENG over LYVE1 were 3.9, 2.1, and 18.0, respectively. In pituitary tumor tissue, those ratios were increased substantially to 8.4, 17.9, and 55.7, respectively. This suggests that the elevated expression of endothelial markers is mainly due to a higher number of vascular endothelial cells, which reflects angiogenesis activity in pituitary tumors. Therefore, we reasoned that the contribution of angiogenic factors to angiogenesis in tumors could be evaluated by analyzing correlations between their expression and the expression of vascular endothelial markers. We performed a correlation analysis with data from NFAs and GH-producing tumors. Among 151 NFAs (**Table 1**), 140 tumor samples were used for analysis because expression data of all 19 genes (**Figures 2**–**5**) were available. For the same reason, 38 out of 39 GH-producing tumors (**Table 1**) were used for the analysis. The sample numbers of ACTH- and PRL-producing tumors were 16 and 13, respectively, which were too small to perform meaningful correlation analyses. The full Pearson’s correlation coefficient estimates for NFA and GH-secreting tumors are included in **Supplementary Figure S1** and **S2**.

**Figure 6:**
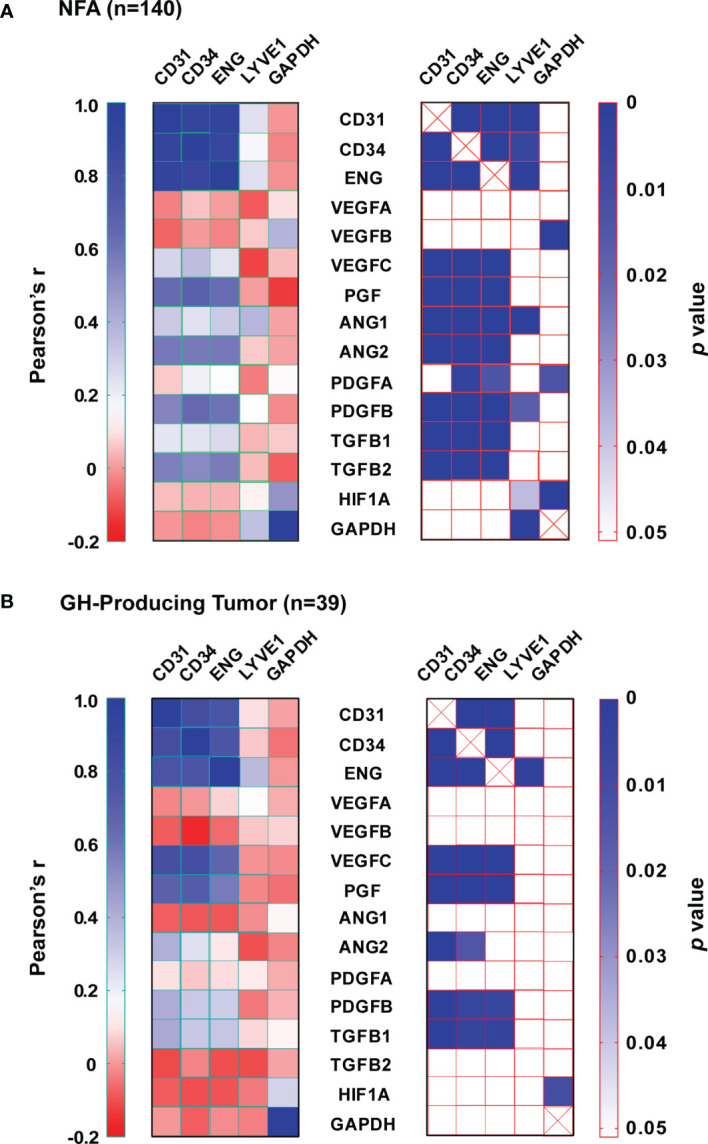
Expression correlation between angiogenic growth factors and endothelial markers. **(A)** NFA and **(B)** GH-secreting tumors. Left panel, Pearson’s correlation coefficients (*r*). Right panel, p value.

In NFAs, CD31, CD34, and ENG levels correlated very strongly with each other (**Figure 6A**). The correlations between their expression and LYVE1 were much weaker. Endothelial marker levels did not correlate with GAPDH levels. Among angiogenic growth factors, PGF, ANGPT2, PDGFB, and TGFB2 correlated most strongly with each of the endothelial marker levels. These are members of four signaling pathways and play essential roles in angiogenesis sprouting and blood vessel maturation. A similar pattern was also observed in GH-producing tumors. Expression of at least one member from each of the four pathways positively correlated with endothelial marker expression (**Figure 6B**). Although both TGFB1 and TGFB2 correlated significantly with endothelial markers in NFAs (**Figure 6A**), only TGFB1, not TGFB2, positively correlated with endothelial markers in GH-producing tumors (**Figure 6B**). Moreover, neither expression of VEGFA nor VEGFB correlated with the expression of any endothelial marker. However, VEGFC expression correlated significantly with endothelial markers in both types of tumors (**Figures 6A, B**). Taken together, these data strongly suggest that human pituitary tumors maintain an active angiogenesis program. These data also suggest that VEGFC and PGF are the main members of VEGF family that regulate angiogenesis in human pituitary tumors.

### Pituitary Tumors Stimulate Angiogenesis *via* the VEGF Signaling Pathway

**Figure 7:**
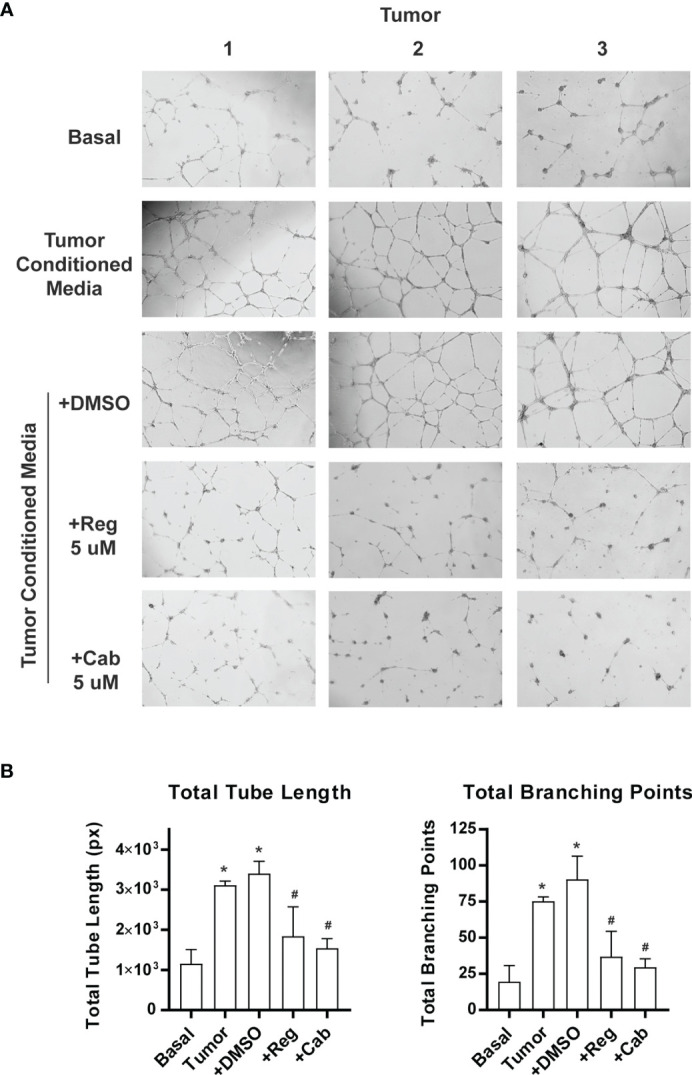
Inhibition of pituitary tumor angiogenesis by VEGF receptor inhibitors. Angiogenesis was assayed by endothelial cell tube formation in Matrigel gel. The pictures were analyzed by WimTube online program. **(A)** A sample picture used for analysis by WimTube. **(B)** Tube formation is presented as total tube length (*left panel*) and total branching points (*right panel*) per field. The values are mean ± SD of experiments with three NFAs. *p < 0.05 vs basal medium; #p < 0.05 vs treatment with conditioned media.

We performed *in vitro* endothelial tube formation assays using culture media conditioned with primary human pituitary tissue. The assay is widely used to identify angiogenic and antiangiogenic factors by mimicking multiple steps in angiogenesis (48). Primary tumor tissue, which consists of both tumor cells and the tumor microenvironment (TME), was used. The TME is composed of non-tumor cells (such as fibroblast, folliculostellate, and immune cells) and extracellular matrix harboring enzymes, growth factors, and cytokines (49, 50). The TME is known to play an important role in tumor angiogenesis (51). Three tumors, including 2 NFAs and 1 GH-producing tumor, were included in the assay because we have observed that both types of tumors contain active angiogenesis programs (**Figure 6**). Media from primary pituitary tumor cultures increased endothelial tube formation in Matrigel by approximately 3-fold, measured as total tube length, and 4-fold as total branching points (**Figures 7A, B**). Regorafenib is an inhibitor of multiple receptor kinases, including VEGF receptors 1, 2, and 3 (52). Cabozantinib predominantly inhibits VEGF receptor 2 (23). When regorafenib (Reg) or cabozantinib (Cab) was added to the conditioned media, tube formation was significantly reduced (**Figures 7A, B**). These experiments suggest that angiogenesis induced by human pituitary tumors is mediated by the VEGF signaling pathway.

### Prolonged Survival by VEGF Receptor Inhibitor Cabozantinib in Mice With Pituitary Tumors

**Figure 8:**
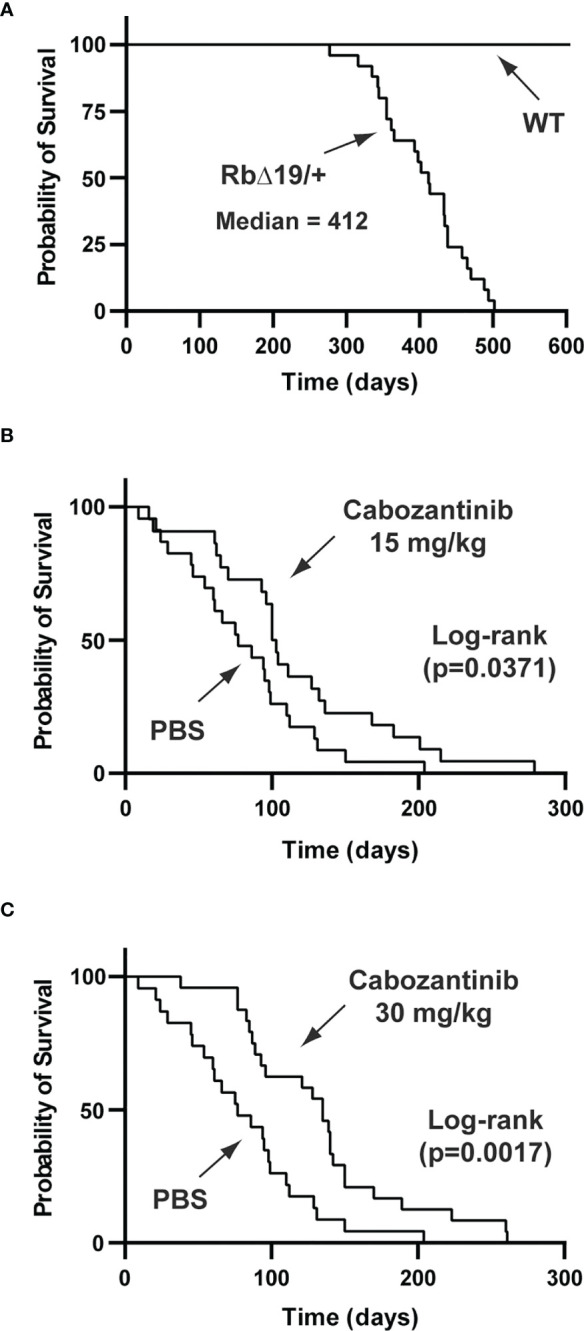
Prolonged survival of *RbΔ19* mice by cabozanitinib treatment. **(A)** Survival of *RbΔ19* mice and their WT littermates in the absence of antiangiogenesis treatment. **(B)** Survival of *RbΔ19* mice treated with Cab at 15 mg/kg body weight for two two-week regimens. Mice treated with PBS were as controls. **(C)** Survival of *RbΔ19* mice treated with Cab at 30 mg/kg body weight for two two-week regimens. The treatment was initiated when mice reached 345 days old. Time designated as days after treatment initiation.

Inhibition of angiogenesis by anti-VEGF antibody or by VEGF receptor inhibitor was tested in prolactinoma mouse models (53–55). To test whether inhibition of angiogenesis suppressed a broader spectrum of pituitary tumors, we chose the Rb knockout mouse as our experimental model. The Rb pathway plays an important role in the pathogenesis of pituitary tumors (56). In humans, the RB pathway is silenced in approximately 90% of pituitary tumors (56). In mice, inactivation of the Rb pathway by deletion of the Rb gene, by overexpression of Rb inactivating oncogenes, or by deletion of CDK inhibitors promotes the development of pituitary tumors (57). For example, overexpression of the SV40 T-antigen in the pituitary gland resulted in GH-producing and null cell adenomas in mice (58, 59). For our study, we created *RbΔ19* mice in which the exon 19 of the *Rb1* gene had been deleted. The *RbΔ19 mice* were derived from *Rb1^tm2Brn^* mice, which carry a floxed exon 19 of the *Rb1* gene (22). We observed that heterozygous RbΔ19 mice all developed pituitary tumors, with a median survival of 412 days (**Figure 8A**). To determine when to initiate antiangiogenesis treatment in *RbΔ19* mice, we analyzed pituitary tumors from a set of apparently healthy mice at 345 days after birth. We observed that 16 out of 18 mice examined had visible pituitary tumors (**Supplementary Figure S3**). Histological staining confirmed that these tumors were adenomas. It also revealed that the other two pituitary glands contained micro tumors. Therefore, we chose 345 days as the age to begin antiangiogenesis treatment.

Three cohorts of *RbΔ19* mice were included in the experiments. Fifteen mg cabozantinib/kg body weight (Cab-15), 30 mg/kg body weight (Cab-30) and PBS, respectively, was administered. The drug has been approved for the treatment of human metastatic medullary thyroid cancer, advanced renal cell carcinoma, and hepatocellular cancer under the trade name COMETRIQ™. The health of all animals was monitored closely. They were euthanized when they became moribund. After euthanasia, causes of death were determined by necropsy. Mice that died of causes not related to pituitary tumors were excluded from the final analysis (see Materials and Methods). The mice in the PBS group (n=23) died between 9 to 204 days after the first dose was administered, with a median survival of 77 days (ranging from 9 to 204 days) after treatment initiation. In contrast, the median survival after the treatment was 101 days (16 to 279 days) for mice in Cab-15 group (n=22, vs PBS group, p = 0.0371) and 135 days (38 to 261 days) for mice in the Cab-30 group (n=24) vs the PBS group p = 0.0017) (**Figures 8B, C**).

There were no apparent adverse effects caused by Cab on wild-type mice. The WT mice administered 30 mg Cab/kg body weight lived well beyond 24 months, except for one mouse euthanized 365 days after treatment initiation. This animal developed a severe eye lesion and had to be euthanized following IACUC rules. A side effect that was only observed in *RbΔ19* mice treated with Cab was the development of grey patches on their fur. These patches slowly disappeared a few months after drug administration.

## Discussion

Our data suggest that human pituitary tumors, regardless of tumor type, are more vascular, have elevated expression of angiogenic growth factors, and upregulate angiogenic pathways more than normal pituitary gland tissue. In addition, we identified PGF and VEGFC as the major angiogenic growth factors regulating angiogenesis in human pituitary tumors. Furthermore, we show that targeting VEGF receptors significantly improves the survival of mice with pituitary tumors. These data provide a rationale for studying whether agents that target these pathways may be effective therapies for aggressive pituitary tumors.

In this study, we systematically quantified the expression of multiple endothelial markers and angiogenic genes in a large cohort of human pituitary adenomas. To the best of our knowledge, this is the first study employing the TaqMan^®^ probe-based absolute qPCR technique to assess the expression of angiogenic genes in human pituitary tumors. One advantage of using the absolute qPCR is that it avoids biases introduced by internal reference genes. Another advantage is that data from absolute qPCR makes it possible to perform correlation analyses to analyze the roles individual angiogenic genes may play in pituitary tumor angiogenesis. In contrast, the large majority of reports in the literature investigating pituitary angiogenesis have examined vascularization by MVD assays. Using this method, some studies demonstrated decreased vascularization of pituitary tumors compared with normal pituitary glands, and others, even within the same papers, were contradictory. For example, Turner et al. demonstrated a lower density of blood vessels in benign pituitary tumors than in normal pituitary tissue; however, macroadenomas demonstrated greater vascularity than microadenomas (17). In contrast, Takada et al. reported that the capillary vessel number per area in pituitary tumors was significantly higher than in normal pituitary gland tissue (60). However, if MVD were measured as the percentage of area, it was lower in the tumor tissue than in normal pituitary gland tissue (60), suggesting that pituitary tumors contain a greater number but smaller capillary vessels. Similarly, Perez-Millan et al. reported that human pituitary tumors contained more small blood vessels than normal pituitary glands (61). Other published studies have used endothelial markers to investigate angiogenesis in pituitary tumors. For example, Rotondo et al. observed a higher MVD in human pituitary tumors than in normal pituitaries when tissue sections were stained with an anti-CD105 antibody (62). CD105 is encoded by the ENG gene, which is believed to be expressed only in proliferating endothelial cells (35). Therefore, some, but not all, prior studies support our conclusion that angiogenesis is more active in human pituitary tumors than in normal pituitary glands.

Sprouting angiogenesis is a well-established mechanism of tumor angiogenesis (12). It is regulated by a signaling cascade of multiple angiogenic pathways involving VEGF, ANGPT2, PDGF, and TGFβ (15, 24). VEGF initiates the sprouting of endothelial cells by stimulating endothelial tip cell transformation and migration (16). ANGPT2 destabilizes vessels by antagonizing ANGPT1 to facilitate tip cell sprouting (30). TGFβ and PDGF signaling contribute to the growth and maturation of new blood vessels (24). Our data demonstrate that human pituitary tumors highly express these angiogenic growth factors (**Figures 3**–**5**). In addition, their expression levels strongly correlate with the expression of vascular endothelial markers (**Figure 6**). Therefore, our data strongly support that sprouting angiogenesis is an angiogenic mechanism for neovascularization in human pituitary tumors. Nevertheless, our data do not exclude the existence of other vascularization mechanisms in human pituitary tumors, such as vasculogenic mimicry (63).

A surprising finding was that expression levels of VEGFA and VEGFB did not correlate with the expression of endothelial markers (**Figure 6**). In contrast, Cristina et al. reported a positive correlation between VEGFA expression and CD31 in NFAs with an R^2^ of 0.49 (p=0.010) (64). The discrepancy may be due to differences in data collection between the two studies. They quantified protein levels using Western blotting, and data were normalized against the internal control gene actin. In contrast, we quantified mRNA levels using absolute qPCR, and data are presented as copy numbers per ug total RNA. Our data indicate that PGF and VEGFC expression correlate strongly with endothelial marker expression (**Figure 6**). This suggests that PGF and VEGFC are the main angiogenic growth factors initiating angiogenesis in human pituitary tumors.

VEGF family of growth factor signaling is mediated by three receptors: VEGFR1 (encoded by the FLT1 gene), VEGR2 (KDR), and VEGFR3 (FLT4). These receptors form homo or heterodimers and are activated by autophosphorylation upon binding to VEGF ligands (29, 65). VEGF1 is predominantly expressed on blood vessel endothelial cells (66), and PGF binds only to VEGF1 (67). In addition to VEGFR2, VEGFA also binds to VEGFR1 with high affinity (67). Because it is characterized by very weak kinase activity, VEGFR1 is thought to be a negative regulator of the VEGF signaling by acting as a decoy receptor (29). We found that its expression in human pituitary tumors is approximately 2-fold that of normal pituitary tissue (FLT1 in **Figure 1**). VEGFR2 is expressed mostly in vascular endothelial cells (68). Both VEGFA and VEGFC bind to VEGFR2. VEGFR2 is the predominant receptor mediating VEGF-induced vascular angiogenesis (29, 68). Consistent with this, we found that VEGFR2 expression is more than 8-fold higher in pituitary tumors than in normal pituitary tissue (KDR in **Figure 3A**). Its expression correlated positively with levels of vascular endothelial markers in both NFAs and GH-secreting tumors (**Supplementary Figures S2** and **S3**), suggesting that VEGFR2 plays an important role in pituitary tumor angiogenesis. VEGFR3 only binds to VEGFC. VEGFC/VEGFR3 normally regulates lymphatic angiogenesis (69). We did not investigate VEGFR3 expression in our study, and its role in pituitary tumor angiogenesis is not known.

In pituitary tumors, PGF may regulate angiogenesis *via* several mechanisms. PGF binds to the VEGFR1 to free VEGFA from its receptor, thereby increasing interactions between VEGFA and VEGFR2 (70). PGF forms a heterodimer with VEGFA, which induces VEGFR1/VEGFR2 receptor dimers, thereby activating VEGFR2 by transphosphorylation (71). Therefore, VEGFA likely stimulates angiogenesis in human pituitary tumors in a PGF-dependent manner. PGF can also stimulate angiogenesis independently of VEGF by activating VEGFR1 (71). VEGFC has long been considered to be the main regulator of lymphangiogenesis (72). However, multiple studies indicate that it also plays an important role in regulating endothelial sprouting (73–75). These data raise the possibility that treatment with the anti-VEGFA antibody, bevacizumab, may not be sufficient to inhibit angiogenesis in pituitary tumors. VEGF-inhibition resistance has been reported in a number of cancers (76), and induction of PGF is believed to be one of the causes (76). Ideally, a panel of angiogenic factors could be assessed in surgically removed tumors and specific antiangiogenesis drugs selected according to the expression profile of the angiogenic growth factors observed in a specific tumor.

Anti-VEGF treatment has been demonstrated to successfully treat pituitary tumors in mouse models. Korsisaari et al. used the anti-VEGF antibody G6-31 to treat pituitary tumors in *Men1^+/-^* mice (53). After 67 days, they observed that G6-31 treatment reduced the tumor volume by 72% compared to the control group and significantly extended their survival. All pituitary tumors in *Men1^+/-^* mice were later identified as prolactinomas (53). The G6-31 antibody was also used to treat Drd2(-/-) mice with hyperplastic pituitary glands by Luque et al. (54). A group of female Drd2(-/-) mice were treated with G6-31 for six weeks (54). Pituitary hyperplasia was significantly reduced at the end of the treatment period compared to the placebo group. A reduction in MVD in G6-31 treated pituitary glands was also observed (54). Axitinib is a VEGF inhibitor targeting all three VEGF receptors (77), and it has been approved by the FDA to treat human renal cell carcinoma. Chauvet et al. tested axitinib on a transgenic mouse model expressing a truncated form of Hmga2 protein (55). Hmga2 mice develop prolactinomas between 9-11 months. Axitinib was administered to animals with a high circulating prolactin level for six weeks. At the end of the treatment period, they observed a significant reduction in tumor size and improvement in vascular structure (55). The main shortcomings of these studies were: 1) all tumors included in the treatment were prolactinomas, and 2) a small number of mice were used. For example, the survival curves after treatment in the study by Korsisaari et al. only included 8 and 9 mice for G6-31 and the control group, respectively (53). However, these studies suggest that the efficacy of such agents may merit investigation for the treatment of aggressive pituitary tumors.

Mice carrying a heterozygous allele of the mutant *Rb1* gene are known to develop anterior pituitary tumors on the C57BL/6 background (78). We found that approximately 70% of *RbΔ19* mice of the 8^th^ or greater generation after backcrossing with C57BL/6 developed anterior pituitary tumors, with about 25% of all tumors expressing ACTH, 25% PRL, 35% GH, 25% TSH, 10% LH and 25% expressing no hormones (data not shown). In addition, some tumors expressed more than one hormone. Therefore, the *RbΔ19* model may be seen as representative of all types of pituitary tumors. We also had a minimum of 22 mice in each treatment group, which is adequate for statistical analysis. Treatment with 15 mg or 30 mg Cab/kg body weight (the latter dose has been used previously to treat cancers in mouse models (23, 79)) for two two-week regimens significantly extended the survival of the *RbΔ19* mice (**Figure 8**). Interestingly, the prolonged survival appeared to be dose-dependent (**Figure 8**). However, there was no statistically significant difference in survival between the two doses. Although two two-week treatments did not seem to have resulted in any side effects in WT mice, we did not investigate whether mice with pituitary tumors could tolerate long-term administration of this agent. Another limitation of our study was our inability to randomize mice with similar tumor sizes to different treatments. Due to the prohibitive cost associated with scanning a large number of mice, it was impossible for us to select mice with tumors of specific sizes. Our goal was to determine whether inhibition of angiogenesis by targeting the VEGF pathway could suppress pituitary tumor growth, and we were able to show that this was the case by demonstrating prolonged survival. We also showed that inhibition of angiogenesis can shrink or slow tumor growth but does not entirely eradicate tumors. However, in practice, tumor eradication is not necessary, as shrinking or even arresting tumor growth can preserve vision, prevent mass effect and is often adequate treatment. Therefore, if such therapy were to achieve these goals in humans with aggressive pituitary tumors, it could have a significant impact.

In summary, our data indicate that human pituitary tumors are characterized by upregulation of angiogenesis compared with normal pituitary glands. Our data also suggest that pituitary tumor angiogenesis may follow the well-established sprouting angiogenesis model. Furthermore, we identified PGF and VEGFC, but not VEGFA or VEGFB, as the major angiogenic growth factors regulating angiogenesis in human pituitary tumors. Finally, we demonstrate that targeting VEGF receptors, thus inhibiting angiogenesis significantly improves the survival of mice with pituitary tumors. Our data suggest that angiogenesis inhibition as a therapy for the treatment of aggressive human pituitary tumors merits further investigation.

## Data Availability Statement

The original contributions presented in the study are included in the article/**Supplementary Material**. Further inquiries can be directed to the corresponding author.

## Ethics Statement

The studies involving human participants were reviewed and approved by Mass General Brigham Institutional Review Board. Written informed consent for participation was not required for this study in accordance with national legislation and institutional requirements. The animal study was reviewed and approved by the Massachusetts General Hospital Institutional Animal Care and Use Committee.

## Author Contributions

YZ and AK conceptualized and designed the study. JZ, YH, WZhu, CN, WZhao, and KL collected experimental data. AF, BS, EH-W, and PJ collected the tumor samples and clinical data. JZ, XZ, HL, XZ, and YZ analyzed and interpreted data. JZ and YZ wrote the first draft manuscript. AF, KM, and RS critically reviewed and edited the manuscript. RJS and AK provided study funding. All authors reviewed the results and approved the final version of the manuscript.

## Funding

This work was supported in part by the National Institutes of Health (R01 CA193520 to AK and RS) and the Jarislowsky Foundation (AK).

## Conflict of Interest

The authors declare that the research was conducted in the absence of any commercial or financial relationships that could be construed as a potential conflict of interest.

## Publisher’s Note

All claims expressed in this article are solely those of the authors and do not necessarily represent those of their affiliated organizations, or those of the publisher, the editors and the reviewers. Any product that may be evaluated in this article, or claim that may be made by its manufacturer, is not guaranteed or endorsed by the publisher.
