# Supplementary material for: Sprouting Angiogenesis in Human Pituitary Adenomas
Source: Front Oncol. 2022 May 5;12:875219. doi: 10.3389/fonc.2022.875219 (PMC9117625; doi:10.3389/fonc.2022.875219)

**Supplementary Figures**

Zhou J. et al. Sprouting Angiogenesis in Human Pituitary Adenomas

**Figure S1**

The Pearson’s correlation analysis between expression of endothelial markers and angiogenic genes in human clinically non-functioning pituitary adenomas. The analysis was performed with data from 140 NFAs. (A) Pearson’s coefficients (r). (B) p values. P values less than 0.001 were not indicated in the graph.


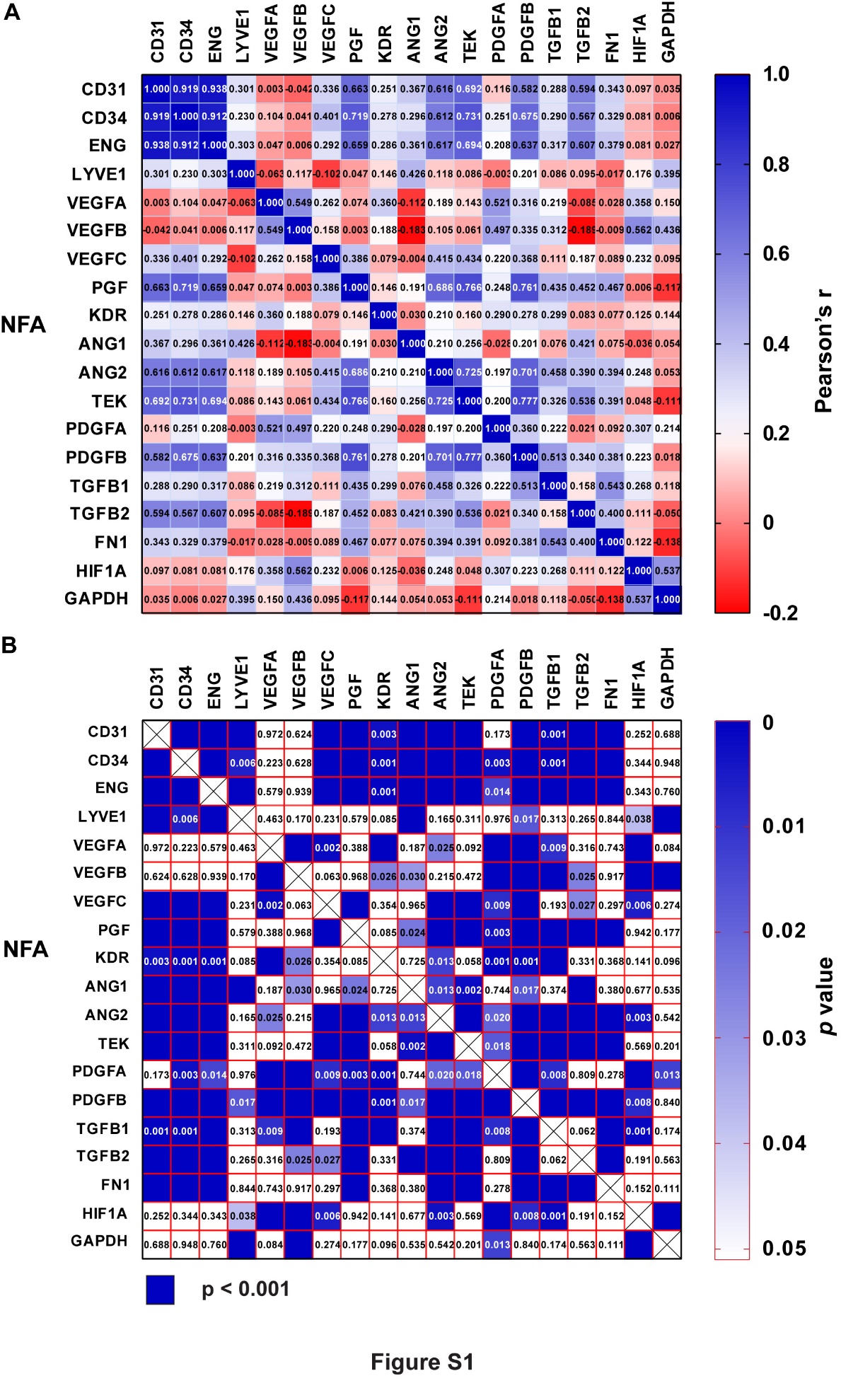


**Figure S2**

The Pearson’s correlation analysis between expression of endothelial markers and angiogenic genes in human GH-secreting pituitary adenomas. The analysis was performed with data from 38 GH-secreting tumors. (A) Pearson’s coefficients (r). (B) p values. P values less than 0.001 were not indicated in the graph.


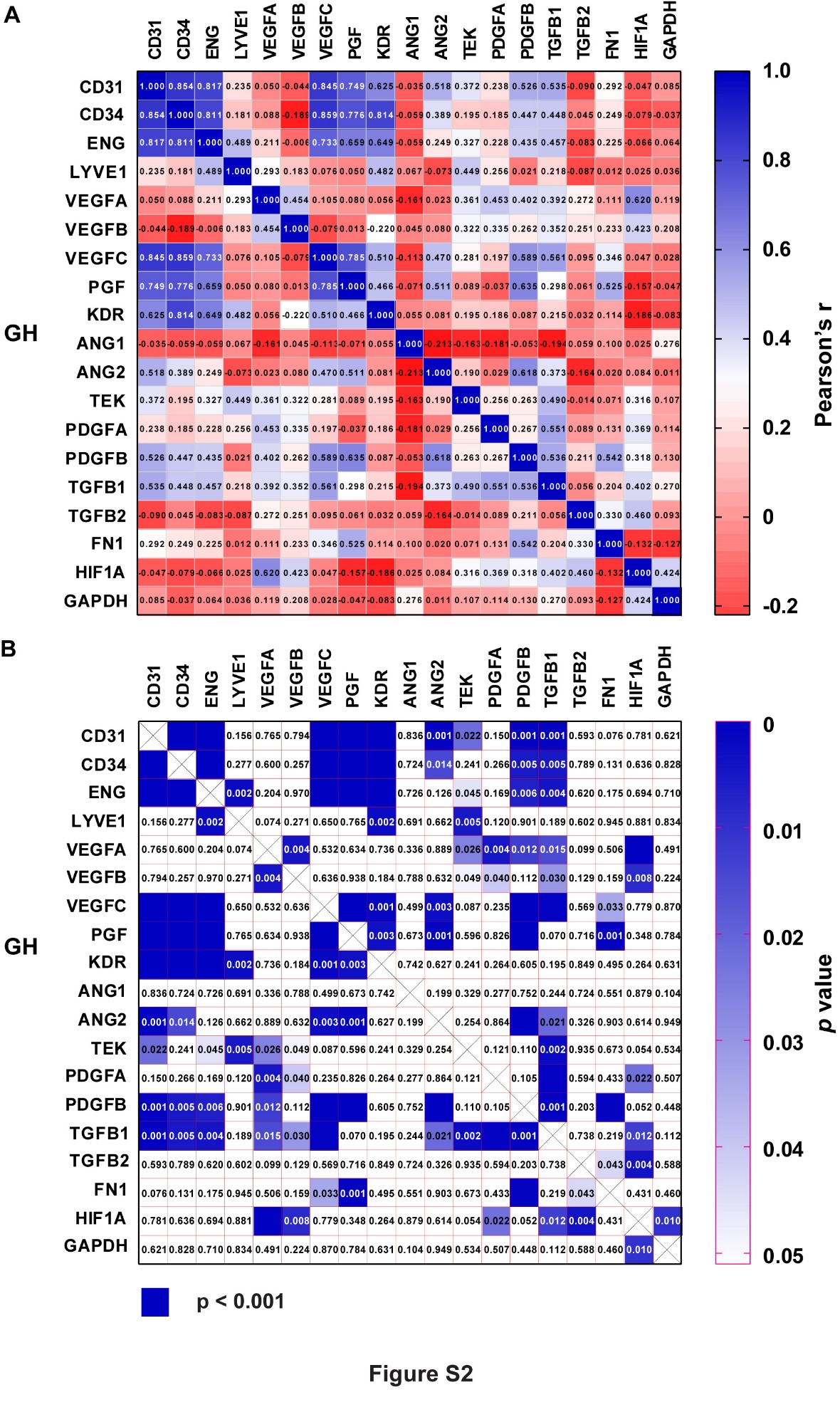


**Figure S3**

Pituitary tumors in *RbΔ19* mice at the age of 345 days. *RbΔ19* mice were euthanized at the specified age. Their pituitaries were exposed and photographed. (A) Pituitaries from 5 WT littermates. (B) Pituitaries from 18 *RbΔ19* mice.


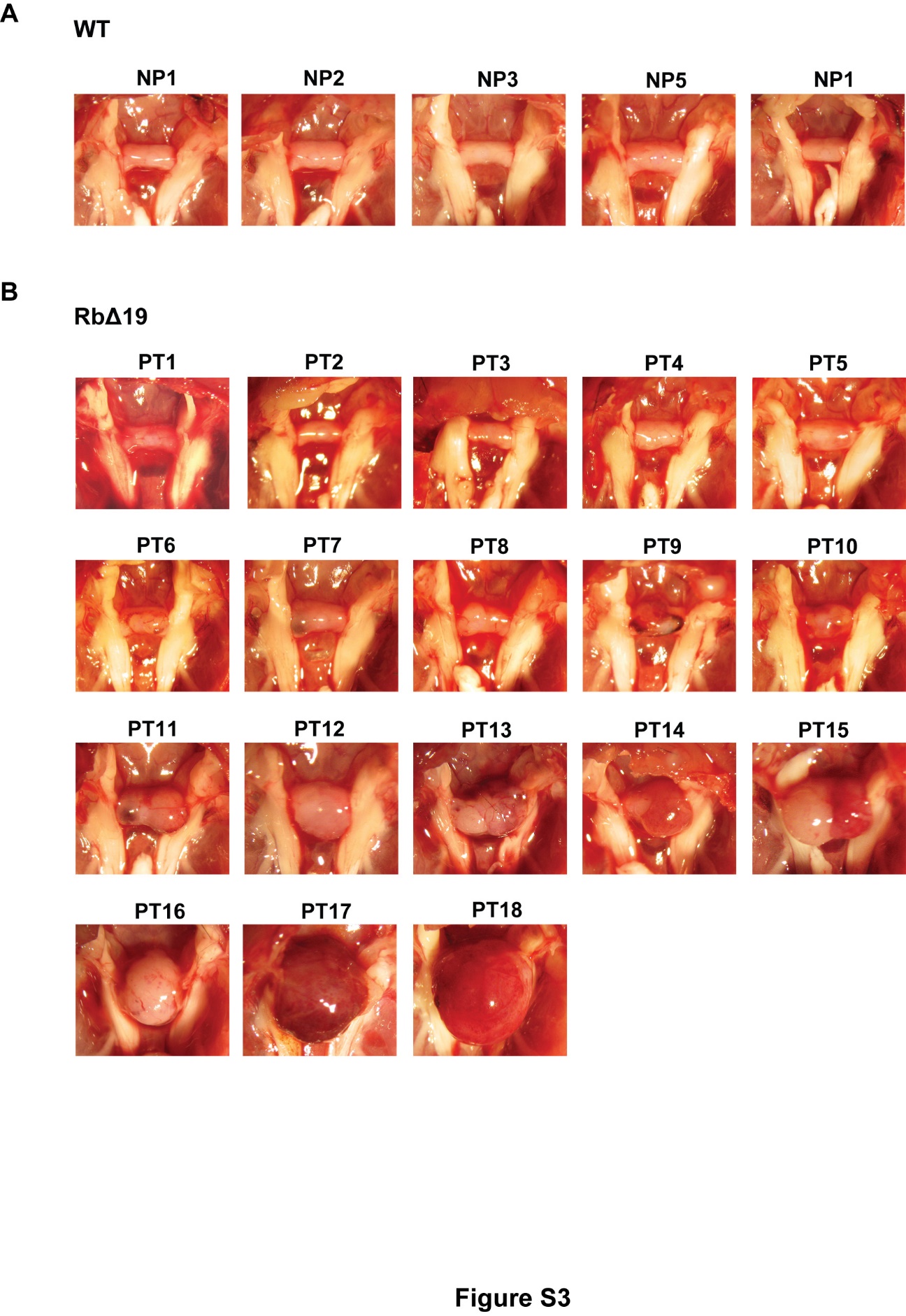

Supplement: Supplementary file 1 [file DataSheet_1.docx]
